# Novel Modelling Approaches to Characterize and Quantify Carryover Effects on Sensory Acceptability

**DOI:** 10.3390/foods7110186

**Published:** 2018-11-08

**Authors:** Damir Dennis Torrico, Wannita Jirangrat, Jing Wang, Penkwan Chompreeda, Sujinda Sriwattana, Witoon Prinyawiwatkul

**Affiliations:** 1 School of Agriculture and Food, Faculty of Veterinary and Agricultural Sciences, The University of Melbourne, Parkville, VIC 3010, Australia; 2 School of Nutrition and Food Sciences, Louisiana State University Agricultural Center, Baton Rouge, LA 70803, USA; Wannita.Jirangrat@thaiunion.com (W.J.); wprinya@lsu.edu (W.P.); 3 College of Nursing and Health Innovation, University of Texas, Arlington, TX 76019, USA; jing.wang@uta.edu; 4 Department of Product Development, Faculty of Agro-Industry, Kasetsart University, Bangkok 10900, Thailand; fagipkc@ku.ac.th; 5 Sensory Evaluation and Consumer Testing Unit, Faculty of Agro-Industry, Chiang Mai University, Chiang Mai 50100, Thailand; sujinda.s@cmu.ac.th

**Keywords:** carryover effects, sensory acceptability, sensory bias, mixed models, nonlinear models

## Abstract

Sensory biases caused by the residual sensations of previously served samples are known as carryover effects (COE). Contrast and convergence effects are the two possible outcomes of carryover. COE can lead to misinterpretations of acceptability, due to the presence of intrinsic psychological/physiological biases. COE on sensory acceptability (hedonic liking) were characterized and quantified using mixed and nonlinear models. *N* = 540 subjects evaluated grape juice samples of different acceptability qualities (A = good, B = medium, C = poor) for the liking of color (C), taste (T), and overall (OL). Three models were used to quantify COE: (1) COE as an interaction effect; (2) COE as a residual effect; (3) COE proportional to the treatment effect. For (1), COE was stronger for C than T and OL, although COE was minimal. For (2), C showed higher estimates (−0.15 to +0.10) of COE than did T and OL (−0.09 to +0.07). COE mainly took the form of convergence. For (3), the absolute proportionality parameter estimate (λ) was higher for C than for T and OL (−0.155 vs. −0.004 to −0.039), which represented −15.46% of its direct treatment effect. Model (3) showed a significant COE for C. COE cannot be ignored as they may lead to the misinterpretation of sensory acceptability results.

## 1. Introduction

The sensory biases caused by the residual sensations of previously served samples are known as carryover effects [1,2,3]. Contrast and convergence effects are the two possible outcomes of carryover effects. The contrast effect is defined as the increased perceived difference or discrepancy among products in a sample set. Conversely, the convergence effect is related to the increased perceived similarity among products [2,4,5,6,7]. The contrast and convergence effects are hypothesized to affect hedonic results differently. Acceptability ratings tend to decrease when a poor-quality product is preceded by a good-quality product (contrast effect). Contrariwise, acceptability ratings for a good-quality product tend to decrease when it is preceded by another good-quality product (convergence effect) [3]. This problem is commonly encountered in crossover trials in which subjects receive a sequence of different sample sets in multiple products assessments. The effect of the first sample usually carries over to the second sample, and this process is repeated for any other subsequent sample in the trial set. In crossover experiments, the carryover effect can lead to misinterpretations when differences among products can increase or decrease inappropriately, due to the presence of intrinsic faults in the experimental design [8]. Besides, carryover can also affect the development of new products, where multiple samples are tested disregarding the potential biases.

The positional (related to the order in which samples are presented) and carryover effects may affect the sensory acceptability ratings of products by confounding the estimated parameters of the sample/treatment effect (the unbiased sensory difference among the tested products) with the intrinsic sample order effect (the psychological bias originated from the experimental procedure). In sensory evaluation, several techniques have been proposed to minimize carryover effects during tasting, including extending washout periods and using different palate cleansers [9,10]. Carrot sticks and sparkling mineral water [11], bread and mineral water [12], Melba toast and neutral water [13], unsalted cracker and soda water [14], apple slices and carbon filtered water [15] are some of the cleansers commonly used in sensory tests. In terms of the experimental plan, Jirangrat et al. [8] demonstrated that the split-plot design is a suitable experimental procedure for reducing order effect biases in multiple products testing. Split-plot designs can achieve outcomes that are less susceptible to bias by extracting a larger portion of the explained variance from the error (unexplained variance). However, the intrinsic carryover effects may affect the scores of sensory attributes in consumer tests even with the use of proper experimental designs (randomized complete block design, and/or split-plot design) and protocols for testing (such as providing sufficient time in between samples). In a previous study, the serving order of red wines with different alcohol concentrations affected the sensory perceptions of panelists [16]. Currently, there are limited published works [2,3,17] regarding the identification and quantification of carryover effects on acceptability tests using untrained naïve consumers (whom, due to a lack of training in sensory practices, can be susceptible to different product and testing biases). Besides, the majority of the research has been conducted with trained assessors (10 to 15 panelists), whom may show less psychological biases when assessing foods or beverages [18] since they have acquired certain familiarity levels with the sensory testing procedures. Therefore, quantifying the presence of carryover effects on untrained consumer panelists is still unclear.

For acceptability tests, hedonic liking scores are used to represent the level of satisfaction of consumers toward samples or prototypes. Analysis of variance (ANOVA) has been extensively used to test whether hedonic means from different samples are significantly different, regardless of the presence of order effects or any other confounding bias. ANOVA is conducted under the assumption that all the responses follow a normal distribution, where a minimum or no confounding effect is involved. However, such an assumption is, in most cases, unfulfilled, since psychological and physiological biases generally affect the assessment of products in a sample set. Our previous study [8] identified and quantified the contrast effects of samples in hedonic ratings using ANOVA and baseline logistic regression. However, knowledge about the contrast and convergence effects on acceptability tests is still very limited. In the present study, several alternative statistical models were further investigated, including generalized linear mixed models and nonlinear mixed models.

For mixed effects models, two types of coefficients are estimated, including fixed (a characteristic of the entire population) and random (a characteristic of individual experimental or observational units) effects. Mixed models can fit the data with correlations where the response is not necessarily normally distributed. These correlations can arise from repeated observations/measurements of the same sampling unit. In consumer tests, a set of samples is assessed, in which the responses (scores) are correlated within each assessor. Thus, mixed models are appropriate for analyzing consumers responses within a given experimental design. On the other hand, nonlinear models can be also used to predict responses that do not necessarily follow a linear function. A nonlinear model is an extension of a generalized nonlinear mixed model in which the conditional mean is a nonlinear function that is added to an inverse link of the linear predictors. Lindstrom & Bates [19] and Davidian & Giltinan [20] proposed the use of nonlinear mixed models for repeated measurements. As stated earlier, consumers tests can be considered as an example of repeated measurements, as individuals/panelists (a random sample from the population of interest) score repeatedly under different experimental conditions. By definition, both models (mixed and nonlinear) can be used to investigating the contrast and convergence effects in consumers tests. Thus, the objective of this study was to characterize and quantify carryover effects (due to the psychological bias of consumers that consists in contrasting two products) on sensory acceptability scores using generalized linear mixed models and nonlinear mixed models.

## 2. Materials and Methods

### 2.1. Materials

Grape juice was selected as the product model, due to its simplicity and commonality within the USA. Fruit juices, in general, are simpler food/beverage models compared to gels, solids, or semi-solid systems, in terms of sensory perception [21]. The liquid model was selected to avoid the presence of extra physiological biases generated from the mastication and swallowing of the samples. A prescreening of 10 local (Baton Rouge, LA, USA) commercial grape juice samples was performed with 30 panelists who regularly consumed grape juice (at least one or two times a week) prior to the actual experiment. The pre-selected criteria were based on five sensory attributes, including purple color, liquid transparency, grape flavor, sweetness, and sourness. The acceptability test (overall liking using a 9-point hedonic scale) was carried out for each grape juice sample. The aim was to classify the level of liking for each product. The three products, having preliminary (*N* = 30) liking scores of 8–9 (good), 6–7 (medium), and 4–5 (poor) were selected for this study. To avoid conflicts of interest, the names of these products were not reported.

The consumer evaluation was conducted in the Sensory Analysis Laboratory, School of Nutrition and Food Sciences, Louisiana State University, Agricultural Center, Baton Rouge, LA, USA. A large focus-group type room equipped with multiple tables was used to conduct the sensory test. The test room (temperature 25 ± 2 °C) was illuminated with cool, natural, fluorescent lights. A total of *N* = 540 consumers (*N* = 269 females and *N* = 271 males; >18 years) from a pool of faculty, staff, and students from Louisiana State University were recruited and pre-screened using the following criteria: (1) regular consumers of grape juice based on self-reported responses, and (2) not having taste/smell disorders and/or kidney/liver problems. The use of human subjects in this research was approved by the Louisiana State University Agricultural Center Institutional Review Board (IRB# HE11-29). Consumers were briefed about the questions, particularly the sensory attributes and their meanings, and sample handling during the evaluation. Products were poured into Propak™ Soufflé clear lidded plastic cups (60 mL) (Independent Marketing Alliance, Houston, TX, USA). Each cup was half filled (30 mL) and labeled with 3-digit random codes (generated from the Random Orders of the Digits table [22]).

A balanced crossover design with three treatments and three periods (positions) was carried out in this experiment. This design was uniform within the sequences in the sense that each treatment appeared the same number of times within each sequence. This design was also uniform within periods, meaning that each treatment appeared the same number of times within each period. The design is balanced in the sense that each treatment preceded every other treatment the same number of times. Table 1 shows the 3 × 3 crossover design. Table 2 lists the input design effects and their classification. Three commercial grape juices were classified into three quality categories (good, medium, and poor) according to preliminary liking scores as shown in Table 2. Participants (*N* = 540) were asked to rate their liking according to two sensory attributes (color and taste), and to rate the overall liking of the grape juice samples using a 9-point hedonic categorical scale (a scale with number and definition) where 1 = extremely dislike, 5 = neither like nor dislike and 9 = extremely like [23]. Three main carryover effects (*n* = 3) were estimated in the experimental design, due to the residual effects of their corresponding juice treatments (good, medium, and poor quality).

### 2.2. Statistical Experiment and Analysis

Three grape juice products were served to each participant using one of the six random serving orders (ABC, ACB, BAC, BCA, or CAB, CBA) so that each order was assigned to 90 participants (6 × 90 = 540 in total); in other words, each sequence order (ABC, ACB, BAC, BCA, CAB, or CBA) was assessed 90 times. All products were tasted in a blinded and unbranded manner. To reduce the presentation protocol errors, each participant was exposed to all products (grape juices) at the same time [24].

Water and unsalted crackers were served as palate neutralizers during the experiment. Re-tasting of products was allowed to refresh participants’ memory only if needed [25]. After tasting, each participant was asked to rate three sensory aspects (color, taste and overall liking) of each product. All hedonic data were analyzed at α = 0.05 using the SAS software 9.4 (SAS Institute Inc. Cary, NC, USA). For fitting the data, three models were considered to identify and quantify the carryover effects as shown below:

**Model 1:**

y*_ijk_* = μ + α*_j_* + ρ*_i_*_(*k*)_ + τ*_d_*_(*j,k*)_ + β*_k_* + ϕ*_jk_* + ε*_ijk_* (*i* = 1… 90, *j* = 1, 2, 3 and *k* = 1, …, 6) (1)

where y*_ijk_* is the response due to participant *i*, position *j*, and sequence *k*. μ is the overall mean. α*_j_* is the fixed effect due to position *j*, subject to Σα*_j_*= 0. ρ*_i_*_(*k*)_ is the random effect due to subject *i* being nested within sequence k, assumed to follow a normal distribution with mean zero and a constant variance. τ*_d_*_(*j,k*)_ is the fixed, direct effect of treatment (grape juice) that is assigned to the *j*th position and *k*th sequence, subject to Στ*_d_*_(*j,k*)_ = 0. β*_k_* is the fixed effect due to sequence *k*, subject to Σβ*_k_* = 0. ϕ*_jk_* is the fixed effect (carryover effect) or the interaction effect α*_j_*τ*_d_*_(*j,k*)_ between treatment τ*_d_*_(*j,k*)_ and position *k*, subject to Σϕ*_jk_* = 0. ε*_ijk_* are independent random errors that are assumed to follow a normal distribution with mean zero and a constant variance. ε*_ijk_* and ρ*_i_*_(*k*)_ are independent.

To quantify the magnitude or size and direction of carryover effects in model (1), the carryover effect ϕ*_jk_* was replaced with γ*_d_*_(*j*−1,*k*)_, the fixed effect of the first-order carryover or residual effect from the treatment assigned to the (*j* − 1)th position of the *k*th sequence [26].

**Model 2:**

y*_ijk_* = μ + α*_j_* + ρ*_i_*_(*k*)_ + τ*_d_*_(*j,k*)_ + β*_k_* + γ*_d_*_(*j−1,k*)_ + ε*_ijk_* (*i* = 1, …, 90, *j* = 1, 2, 3 and *k* = 1, …, 6) (2)

where γ*_d_*_(*j−*1*,k*)_ was the fixed effect of the first-order carryover or residual effect from the treatment assigned to the (*j* − 1)th position of the *k*th sequence, subject to Σγ*_d_*_(*j*−1,*k*)_ = 0. Note that there was no carryover effect of the treatment assigned to the first position in each sequence, i.e., γ*_d_*_(0,*k*)_ = 0.

Ferris et al. [2] suggested a proportional relationship between the treatment carryover effect and its direct effect, such that γ*_d_*_(*j*−1,*k*)_ = λτ*_d_*_(*j,k*)_, where |λ| < 1 in general, i.e., model (2) becomes model (3).

**Model 3:**

y*_ijk_* = μ + α*_j_* + ρ*_i_*_(*k*)_ + τ*_d_*_(*j,k*)_ + β*_k_* + λτ*_d_*_(*j,k*)_ + ε*_ijk_* (*i* = 1, …, 90, *j* = 1, 2, 3 and *k* = 1, …, 6) (3)

The sign of λ indicates the form of carryover. When λ > 0, there is an assimilation of the previous treatment, and when λ < 0, carryover takes the form of a contrast effect.

## 3. Results and Discussion

Table 3 presented the type I test of fixed effects estimated from model (1) for three sensory attributes (color, taste, and overall liking). For all sensory attributes, the treatment effect was significant (*p* < 0.0001), indicating that the grape juice samples (A, B, and C) were different in their hedonic scores. As expected, grape juice A had a higher liking score for color, taste, and overall liking (7.36–7.74) than B (6.37–6.71) and C (3.50–3.90) (Table 4). In addition, the position effect (left = 1, center = 2, and right = 3) was significant for taste (*p* = 0.02), but not significant for color (*p* = 0.31) and overall liking (*p* = 0.24). For all attributes (color, taste, and overall liking), the sequence and carryover effects were not significant (*p* ≥ 0.05). However, the carryover effect was stronger for color (*F*-value = 1.94) than for taste (*F*-value = 0.63) and overall liking (*F*-value = 0.59).

In consumer sensory trials, the first order and carryover effects can be minimized by balancing the order of sample presentations when each treatment occurs an equal number of times in each position [27]. Suitable experimental designs such as cross-over (change-over) and Williams designs can be used for minimizing the effects of carryover between samples [28]. In the present study, a Balanced Randomized Block Design (BRBD) was carried out, in which each sample occurs an equal number of times in each position of the trial. Moreover, the 3 × 3 crossover design ensured that all possible adjacent pair of treatments (AB, BA; AC, CA; BC, CB) occurred an equal number of times. The last position (right) had a significantly (*p* < 0.05) lower liking score compared to the first (left) and center positions (5.7 vs. 5.9–6.0 scores for taste; data not shown). The order of tasting can introduce a significant bias in sensory evaluations [28]. In the case of taste, the lower scores reported for the last position (right) in the present study may be partially explained by a common psychological bias of consumers, in which the first sample often receives the highest score in a sequence of samples [29]. However, order and carryover effects are dependent on the sensory context including the nature of the product, attribute selection, and the training level of panelists [30].

Model (2) had the advantage of measuring the magnitude and direction of the treatment carryover effect. In this model, the liking of color showed higher absolute mean estimates of the carryover effect size (−0.15 to +0.10) than did the liking of taste (−0.10 to +0.07) and the overall liking (−0.09 to +0.07) (Table 5), although none of them were significant. The carryover effect represented the size of the bias that can affect the score of the following sample in the sequence. For instance, if the size of the effect was +0.10, this means that the following sample would be positively biased by 0.10 units in the hedonic score. Furthermore, on the 9-point hedonic scale, this carryover effect size +0.10 accounted for (0.1/8) × 100% = 1.25% (considering that the length of the 9-point hedonic scale is 8 units) of its direct treatment effect. In fact, the liking of color had the higher carryover effect bias (−1.90% to 1.29%) than did the liking of taste (−1.26% to 0.87%) and overall liking (−1.15% to 0.82%).

The carryover effects can be classified as contrast and convergence effects, depending on the valence (sign) of the carryover effects (positive and negative), and the intensity of liking (high, medium, or low liking scores) of the previous and current samples tested by panelists. For example, Table 5 showed a positive carryover effect of treatment A for color as +0.10. In Table 4, the rank of the liking scores for the 3 grape juices was A, B, and C for all sensory attributes. If sample A was followed by sample C in the sequence, the consequence of the positive carryover effect of A would be the inflation of the liking of sample C, and therefore the treatment difference would be smaller than if it had been presented in the first position. In this case, the positive carryover effect (+0.10) of A would be classified as the convergence effect. On the other hand, Table 5 shows a negative carryover effect of sample B for color as −0.15. If sample B was followed by sample C in the sequence, then the consequence of the negative carryover effect of B would be a decrease in the liking of sample C, and therefore the treatment difference would be greater than if it had been presented in the first position. In this case, the negative carryover effect (−0.15) of treatment A would be classified as the contrast effect. Table 6 shows the classification of the carryover effects for the treatments (A, B, and C) and attributes (color, taste, and overall) as evaluated in this study.

Carryover was found to affect the descriptive scores of negative attributes in cheeses [28]. In a descriptive analysis of restructured steaks with 15 sensory attributes, Schlich [30] reported that greasiness, tenderness, and juiciness were affected by the carryover effects of previous samples. Conversely, previous studies found no evidence of carryover effects in beverage products [31,32]. Among the treatment effect for all evaluated attributes (color, taste, and overall liking), the visual attribute (color) may hae been largely responsible for the differences among grape juice samples. Color and visual cues of the samples can affect the expectations of consumer and generate contrast and assimilation (convergence) effects if the product does not match the initial expectations [33]. In previous studies, color has been identified as an attribute that can change taste and flavor perception [34,35]. Color is the first attribute that is evaluated by consumers, and it may have the greatest exposure to carryover effects, since it is the attribute that is initially compared to the previous sample.

The sensory responses in the present study were significantly (*p* < 0.05) and positively correlated ((color-taste, *r* = 0.74), (color-overall, *r* = 0.79), (taste-overall, *r* = 0.96); data not shown). Highly positive correlations among sensory attributes are not uncommon in sensory trials (known as Halo effect). This psychological bias has been also studied [1]. However, acknowledging that the attribute responses may be correlated, the present study showed a useful method to characterize and quantify the carryover effects using the mixed linear model (2).

For model (3), the absolute value of the proportionality parameter estimate (λ) was higher for color than for taste and overall liking (0.16 vs. 0.004 to −0.04) (Table 7). The carryover effect for color was significant (confidence intervals of −0.21 and −0.10). The treatment carryover effect for color was higher (−15.46%) than for taste and overall liking (−0.38 to −3.85%). For all attributes, the proportionality parameter (λ) values were negative; thus, the carryover effect mainly took the form of contrast.

Models (1)–(3) showed that the treatment carryover effects for color were higher than for taste and overall liking. Only model (3) showed a significant carryover effect (for color). Although there was some disagreement between models (2) and (3) on the form (convergence vs. contrast) of the carryover effects for taste and overall liking, the carryover effects for these attributes were not significant. However, these generated models are useful tools that can be applied to different food and beverage products in sensory trials that use cross-over and Williams experimental designs. Nonetheless, carryover effects cannot be ignored as it may lead to misinterpretation of the results [18]. The effect that a treatment might have on the assessment of the next treatment (carryover) is more likely to occur when using inexperienced consumers than when using trained panelists [18].

Although models (1) and (2) are based on the typical linear mixed models to account for carryover effects, it should be noted that these models treated the ordinal rating variable as continuous and therefore may be subject to a loss of information between the dependent and independent variables. Despite this disadvantage, Long and Freese [36] agreed that ordinal variables are often treated as being continuous for specific experimental designs. Other alternatives such as the contingency table analysis, cumulative logistic regression, and non-parametric methods can be used for evaluating the ordinal dependent variables, although they have limitations such as a strong requirement of proportional odds across all categories, which is often difficult to meet. Unfortunately, these approaches are not readily available in the context of experimental designs. The great advantage of employing linear model approaches in the context of using the appropriate experimental design (cross-over and Williams) may be enough to offset any disadvantages with which they are accompanied. Moreover, the present study may be subject to measurement errors resulting from the participant’s judgment bias. Measurement errors in dependent variables do not bias the regression estimate but may increase the standard error of the estimate, which in turn may decrease the power. Fortunately, our large sample size of N = 540 may be helpful to offset this issue.

## 4. Conclusions

Consumer tests are prone to carryover effects where responses of samples being evaluated are affected by previously assessed samples. Although there are some published works regarding the quantification of carryover effects associated with trained assessors, quantifying carryover effects on consumer panelists is still unclear. This study characterized and quantified carryover effects on sensory acceptability of grape juices using mixed and nonlinear models. Results from this study showed that color presented a weak carryover effect among the grape juice samples. Besides, this study also proposed useful modeling tools to characterize and quantify the carryover effects in sensory trials using untrained consumers. Further studies are needed to understand the full extent of carryover effects in different food/beverage products and sensory attributes using consumer panels.

## Figures and Tables

**Table 1:** A three-period (position) crossover design.

	Subject	Position		
		1	2	3
Sequence ABC	1	y_1,111_ (A)	y_1,121_ (B)	y_1,131_ (C)
	2	y_2,111_ (A)	y_2,121_ (B)	y_2,131_ (C)
	.			
	.			
	90	y_90,111_ (A)	y_90_,_121_ (B)	y_90,131_ (C)
Sequence BCA	1	y_1,112_ (B)	y_1,122_ (C)	y_1,132_ (A)
	2	y_2,112_ (B)	y_2,122_ (C)	y_2,132_ (A)
	.			
	.			
	90	y_90,112_ (B)	y_90,122_ (C)	y_90,132_ (A)
Sequence CAB	1			
	.			
	.			
	90			
Sequence CBA	1			
	.			
	.			
	90			
Sequence ACB	1			
	.			
	.			
	90			
Sequence BAC	1			
	.			
	.			
	90			

**Table 2:** Description of the input design effect for the grape juice consumer test.

Input Design Effect	Description	Classification
Treatments/Samples (3)	A (good), B (medium), C (poor) ^1^	Fixed
Sequences (6)	ABC, ACB, BAC, BCA, CAB, CBA	Fixed
Sample position (3)	1 (left), 2 (center), 3 (right)	Fixed
Panelists (540)	90 panelists per sequence	Random
Carryover effects (3)	Carryover effect of each treatment	Fixed

^1^ Product quality from a hedonic classification: liking scores of 8–9 (good), 6–7 (medium) and 4–5 (poor).

**Table 3:** Type I test of fixed effects evaluated on three attributes (color, taste, and overall liking) from model (1).

Attribute	Effect	Num DF ^1^	Den DF ^1^	*F*-Value ^2^	Pr > *F* ^2^
**Color**	Sequence	5	534	2.00	0.0778
	Position	2	1074	1.18	0.3072
	Treatment	2	1074	1180.95	*<0.0001*
	Carryover	2	1074	1.94	0.1440
**Taste**	Sequence	5	534	1.60	0.1595
	Position	2	1074	3.91	*0.0204*
	Treatment	2	1074	558.52	*<0.0001*
	Carryover	2	1074	0.63	0.5333
**Overall Liking**	Sequence	5	534	1.15	0.3328
	Position	2	1074	1.43	0.2388
	Treatment	2	1074	706.40	*<0.0001*
	Carryover	2	1074	0.59	0.5549

^1^ Num DF = Degrees of freedom of numerator, Den DF = Degrees of freedom of denominator. ^2^ F-value = Mean square/Mean square error. *F*-value under the hypothesis of Ho: Effect = 0. Effects were considered significant when the probability Pr > *F* was less than 0.05 (Bolded and italicized probabilities).

**Table 4:** Mean values ^1^ for the sensory acceptability scores of the grape juice samples from model (1).

Samples ^2^	Color	Taste	Overall Liking
A	7.36 ± 1.49 ^a^	7.74 ± 1.30 ^a^	7.40 ± 1.45 ^a^
B	6.71 ± 1.32 ^b^	6.37 ± 1.75 ^b^	6.37 ± 1.60 ^b^
C	3.50 ± 1.75 ^c^	3.90 ± 2.08 ^c^	3.74 ± 1.92 ^c^

^1^ Data are represented as mean and standard deviation values (*N* = 540). Liking scores were based on a 9-point hedonic scale (1 = dislike extremely, 9 = like extremely). ^2^ Samples descriptions are shown in Table 2. ^a–c^ mean values with different letters within the same column for each parameter are significantly different (*p* < 0.05).

**Table 5:** Estimates of the carryover effects for different treatments on three attributes (color, taste, and overall liking) using model (2).

Attribute	Parameters ^1^	Carryover Effect for Treatment A	Carryover Effect for Treatment B	Carryover Effect for Treatment C
**Color**	Estimate	+0.10	−0.15	+0.05
	SE	0.08	0.08	0.08
	DF	1074	1074	1074
	*t*-value	1.31	−1.93	0.62
	Pr > |t|	0.19	0.05	0.53
	% of the scale ^2^	+1.29%	−1.90%	+0.61%
**Taste**	Estimate	+0.03	+0.07	−0.10
	SE	0.09	0.09	0.09
	DF	1074	1074	1074
	*t*-value	0.34	0.76	−1.09
	Pr > |*t*|	0.74	0.45	0.27
	% of the scale ^2^	+0.39%	+0.87%	−1.26%
**Overall Liking**	Estimate	+0.03	+0.07	−0.09
	SE	0.09	0.09	0.09
	DF	1074	1074	1074
	*t*-value	0.30	0.75	−1.05
	Pr > |*t*|	0.76	0.45	0.29
	% of the scale ^2^	+0.33%	+0.82%	−1.15%

^1^ SE = Standard error. DF = Degrees of freedom. *t*-value under the null hypothesis of Ho: Estimate = 0. ^2^ % of the Scale = Percentage of the estimate on a 9-point Hedonic Scale (Estimate × 100/8).

**Table 6:** Characterization of the carryover effects on three attributes (color, taste, and overall liking) from model (2).

Previous Sample	Present Sample	Carryover Effects		
		Effect on Color	Effect on Taste	Effect on Overall Liking
A	B	Convergence	Convergence	Convergence
A	C	Convergence	Convergence	Convergence
B	A	Convergence	Contrast	Contrast
B	C	Contrast	Convergence	Convergence
C	A	Contrast	Convergence	Convergence
C	B	Contrast	Convergence	Convergence

**Table 7:** Estimates of the proportionality parameter (λ) for the carryover effects on three attributes (color, taste, overall liking) from model (3).

Attribute	Estimate (λ)	SE ^1^	Approximate 95% Confidence Limits		% of Treatment Effect ^2^
**Color**	−0.16	0.03	−0.21	−0.10	−15.46%
**Taste**	−0.004	0.03	−0.06	0.05	−0.38%
**Overall liking**	−0.04	0.03	−0.09	0.01	−3.85%

^1^ SE = Standard Error. According to Ferris et al. [2], when the estimated λ is positive there is an assimilation of the previous stimulus, while for negative λ carryover takes the form of a contrast effect. The model (*N* = 540) was fitted using PROC NLIN of the SAS software. ^2^ % of treatment effect is the percentage of the carryover effect with respect to the treatment effect in the model.
